# A Child Who Suddenly Freezes While Trying to Cross Crosswalks—Unique Clinical Manifestation of Paroxysmal Kinesigenic Dyskinesia: A Case Report

**DOI:** 10.3390/children7120290

**Published:** 2020-12-14

**Authors:** Sae Yoon Kim, JiYoung Ahn, Soyoung Kwak, Min Cheol Chang

**Affiliations:** 1 Department of Pediatrics, College of Medicine, Yeungnam University, Daegu 38541, Korea; sysnow88@hanmail.net (S.Y.K.); jy4413@gmail.com (J.A.); 2 Department of Physical Medicine and Rehabilitation, College of Medicine, Yeungnam University, Daegu 38541, Korea; july0025@hanmail.net

**Keywords:** paroxysmal kinesigenic dyskinesia, next-generation sequencing, proline-rich transmembrane protein 2, antiepileptic, case report

## Abstract

(1) Background: We report the case of a patient with a unique clinical presentation of inability to cross crosswalks due to paroxysmal kinesigenic dyskinesia (PKD). (2) Case presentation: A 14-year-old boy presented with the inability to move his right leg at gait initiation from the standing position. This episode lasted for approximately 20–30 s and manifested 1–3 times a day. The difficulty in gait initiation usually occurred when the patient tried to cross crosswalks when the traffic light turned from red to blue. His right arm stiffened occasionally while trying to write with a pencil and eat food with a spoon or chopsticks. Other neurological manifestations and pain were absent during these episodes. No neurological symptoms were observed between the attacks. Brain magnetic resonance imaging did not reveal any abnormalities. A next-generation sequencing study revealed a pathological variant in the proline-rich transmembrane protein 2 (*PRRT2*) gene. The patient was diagnosed with PKD. His symptoms disappeared completely after treatment with carbamazepine (100 mg/day). (3) Conclusions: The symptoms of PKD can be successfully controlled using antiepileptic medications. Therefore, clinicians should be aware of the clinical manifestations of PKD to provide appropriate treatment.

## 1. Introduction

Clinicians may frequently encounter pediatric movement disorders in clinical practice [1]. Accurate diagnosis of movement disorders is sometimes difficult owing to the variety of causative underlying diseases and the low incidence of several movement disorders [1]. Paroxysmal kinesigenic dyskinesia (PKD) is a rare episodic movement disorder with an incidence rate of approximately 1 per 150,000 population [2]. The age of onset is between 1 and 20 years. PKD is characterized by sudden episodes of involuntary movements such as dystonia, choreoathetosis, and ballism, lasting for less than 1 min [3]. Loss of consciousness does not occur during attacks, and patients with PKD are neurologically sound between episodes. The episodic abnormal movements are triggered by sudden voluntary movements [3]. The diagnosis of PKD is usually delayed owing to variations in the clinical manifestation and lack of knowledge. The average time to diagnosis is almost 5 years [3]. Therefore, clinicians should have adequate knowledge of the clinical presentations of PKD to prevent misdiagnosis.

In the present study, we describe the case of a patient with PKD with a unique clinical presentation.

## 2. Case Report

A 14-year-old right-handed boy visited the rehabilitation department of a university hospital with a 1-year history of slowly progressive gait difficulty. He had no history of neurological or psychological disorders. The episodes occurred when the patient tried to start walking while standing; he could not move his right leg at gait initiation but could move it after approximately 20–30 s, after which his gait became normal. These episodes occurred 1–3 times a day. This difficulty in gait initiation usually occurred when he tried to cross crosswalks when the traffic light turned from red to blue. His right arm occasionally stiffened while trying to write with a pencil and eat food with a spoon or chopsticks. Other neurological manifestations and pain did not occur during these episodes. No other neurological symptoms were observed between the episodes. Brain magnetic resonance imaging did not reveal any abnormalities.

We speculated that the patient’s symptoms represented a type of dystonia based on the following definition: “Dystonia is a neurological hyperkinetic movement disorder syndrome in which sustained or repetitive muscle contractions result in twisting and repetitive movements or abnormal fixed postures” [4]. A next-generation sequencing (NGS) dystonia panel was conducted for the accurate diagnosis and identification of genes that may be possibly correlated with the underlying disorder. The NGS results revealed a pathological variant in the proline-rich transmembrane protein 2 (*PRRT2*) gene (c.649dupC mutation). *PRRT2* mutations cause PKD and benign familial infantile seizures [5]. We enquired about the existence of similar symptoms in his other family members. The patient’s older brother had similar symptoms in his right arm, which occurred occasionally at a lower intensity. The older brother also had a pathological variant in the *PRRT2* gene (c.649dupC mutation). The younger sister of the patient’s mother (the patient’s aunt) had a history of epilepsy during infancy, but a gene mutation test was not performed.

We diagnosed PKD on the basis of the clinical criteria for PKD (Table 1). Accordingly, the patient received oral administration of carbamazepine (100 mg/day). His paroxysmal movements were well-controlled with no further PKD attack at the 3-month follow-up after treatment initiation.

## 3. Discussion

We made a final diagnosis of PKD based on the clinical criteria for PKD (Table 1) [3] and the results of the NGS study. Our patient showed a good therapeutic response to carbamazepine (100 mg/day).

Patients with PKD present with variable clinical manifestations. PKD episodes are characterized by sudden voluntary movement-triggered abnormal movements such as dystonia, chorea, athetosis, or a combination thereof [3]. When children present abnormal movements (one of the above) in less than 1 min, followed by sudden voluntary movements without alteration of consciousness, clinicians should consider the possibility of PKD, attempt anticonvulsant therapy, and observe the response to medication in children. Our patient had dystonia with his right leg, especially during gait initiation. Psychological anxiety or concerns about traffic accidents could explain why his symptoms presented particularly while crossing crosswalks. Stress is one of the factors that can trigger abnormal movement [3,6].

In 2011, Chen et al. first reported that *PRRT2* gene mutations were the genetic cause of PKD [7]. *PRRT2* protein functions are not fully elucidated. *PRRT2* is expressed in the central nervous system during the embryonic and postnatal stages of development [8,9]. *PRRT2* is known to interact with synaptosomal-associated protein 25 (SNAP25), which fuses synaptic vesicles to the presynaptic plasma membrane and releases neurotransmitters. Moreover, SNAP25 modulates the kinetics of voltage-gated Ca^2+^ channels. The interaction between *PRRT2* and SNAP25 may be affected in patients with *PRRT2* mutations, resulting in dysfunction of the Ca^2+^ channels. This suggests that PKD could result from the dysfunction of a protein involved in synaptic regulation, resulting in neuronal hyperexcitability [10,11].

Additionally, other than *PRRT2*, several genes are related to PKD. Therefore, when patients with symptoms of PKD test negative for *PRRT2* mutation, mutations in other genes including *CAN8A*, *DEPDC5*, *KCNA1*, *KCNMA1*, *PNKD*, and *SLC2A1*, should be considered [12].

Herein, we report the case of a patient with a unique presentation of PKD. Its pathogenesis correlated with mutations in the *PRRT2* gene. Clinicians should be aware of the clinical symptoms of PKD. The diagnosis of PKD should not be missed clinically just because it responds well to anticonvulsant therapy.

## Figures and Tables

**Table 1:** Clinical criteria for the diagnosis of paroxysmal kinesigenic dyskinesia [3].

Clinical Criteria
(1) Identification of the episodic kinesigenic triggers
(2) Short duration of attacks (<1 min)
(3) No loss of consciousness or pain during episodes
(4) Exclusion of other organic diseases and normal neurological findings in the case of primary paroxysmal kinesigenic dyskinesia
(5) Control of the episodes with phenytoin or carbamazepine, if attempted
(6) Age of onset between 1 and 20 years in case of a negative family history of PKD
Moreover, molecular genetic screening for the *PRRT2* gene may be useful for confirming the diagnosis.
